# High Dynamics of Ciliate Community Revealed via Short-Term, High-Frequency Sampling in a Subtropical Estuarine Ecosystem

**DOI:** 10.3389/fmicb.2022.797638

**Published:** 2022-02-07

**Authors:** Bowei Gu, Hungchia Huang, Yizhe Zhang, Ran Li, Lei Wang, Ying Wang, Jia Sun, Jianning Wang, Rui Zhang, Nianzhi Jiao, Dapeng Xu

**Affiliations:** ^1^State Key Laboratory of Marine Environmental Science, College of Ocean and Earth Sciences, Institute of Marine Microbes and Ecospheres, Xiamen University, Xiamen, China; ^2^Fujian Key Laboratory of Marine Carbon Sequestration, Xiamen University, Xiamen, China; ^3^Key Laboratory of Tropical Marine Bio-resources and Ecology, South China Sea Institute of Oceanology, Chinese Academy of Sciences, Guangzhou, China; ^4^College of the Environment and Ecology, Xiamen University, Xiamen, China

**Keywords:** community composition, time series, temporal changes, quantitative protargol stain, microzooplankton

## Abstract

Ciliates are pivotal components of the marine microbial food web, exerting profound impacts on oceanic biogeochemical cycling. However, the temporal dynamics of ciliate assemblages on a short time scale in the highly fluctuating estuarine ecosystem remain largely unexplored. We studied changes in the ciliate community during a short time frame in the high salinity waters (>26) of a subtropical estuary. Ciliate abundance, biomass, size and oral diameter structure, and community composition fluctuated considerably and irregularly over a few days or even a few hours. Spearman correlations and the generalized linear model revealed that heterotrophic prokaryotes (HPs) and viral abundances drove the dynamics of ciliate abundance and biomass. The structural equation model further identified a major path from the high-fluorescence content virus (HFV) to HPs and then ciliates. Given the substantial correlation between salinity and HPs/HFV, we proposed that the response of HPs and HFV to salinity drives the dynamics of ciliate biomass. Additionally, the Mantel test showed that phytoplankton pigments such as Lutein and Neoxanthin, phosphate, and pigmented picoeukaryotes were key covariates of the ciliate community composition. This study demonstrated the highly changing patterns of ciliate assemblages and identified potential processes regulating ciliate biomass and community composition on short timescales in a subtropical, hydrographically complex estuary.

## Introduction

Typically, microbial communities drive marine ecosystem functions (Fenchel, 2008; Worden et al., 2015). Ciliates are a crucial component of marine microbial communities, integrating smaller prey (e.g., prokaryotes and nanoflagellates) and larger zooplanktons by channeling elements to higher trophic levels (Fenchel, 2008). Additionally, ciliate exudates may contain both labile and recalcitrant dissolved organic matter, contributing to the global carbon cycle (Strom et al., 1997; Stoecker, 1999; Jiao et al., 2010). These roles are especially critical in estuarine ecosystems, which harbor rich ciliate prey and many multicellular ciliate grazers (Stoecker and Capuzzo, 1990).

Estuarine systems characterized by strong and periodic tidal fluctuations are profoundly influenced by saltwater-freshwater mixing and tidal pumping (Kowalik, 2004; Heiss and Michael, 2014). Environmental parameters in the estuarine ecosystem typically fluctuate over short time scales, e.g., daily or even hourly. Together with increasing anthropogenic activity, such as the discharge of pollutants (e.g., heavy metals and nutrients), tidal mixing enables estuarine ecosystems to support vital and complex ecological niches and harbor diverse microbial groups that adapt to the highly dynamic estuarine environments (Maurice et al., 2011). The importance of ciliates in marine environments is widely recognized, and studies on ciliates in estuaries have made significant progress as well (Calbet and Landry, 2004). For example, using techniques with high taxonomic resolution, *in situ* investigations have advanced our understanding of the diversity, distribution patterns, and potential driving mechanisms of estuarine ciliates, highlighting the influence of the water mass (as defined by salinity and temperature) and other environmental factors on the ciliate community (Sun et al., 2017, 2020, 2021; Yang et al., 2020; Gu et al., 2021). Due to their small size and fast growth rate, microbial communities cannot remain static throughout time and can respond rapidly to even mild perturbations (Faust et al., 2015). Typically, the abundances of microbial members change considerably between measured time points (Gonze et al., 2018). For example, monthly or interannual time-series studies have shown that the abundance of ciliates in the Bahía Blanca Estuary can fluctuate by an order of magnitude, and wind-induced processes and water turbidity are responsible for ciliate dynamics (Loìpez-Abbate et al., 2019). While these studies yielded valuable insights into the long-term dynamics of ciliates, they could be easily obscured by the strong seasonal variations, particularly in the case of an uneven sampling distribution and subsequent inaccuracies. Additionally, understanding the dynamic patterns and environmental driving factors of microbial communities on a short time scale is crucial for inferring their distribution, activity, and roles in biogeochemical cycling (Khandeparker et al., 2017; Chen et al., 2019). Thus, high-resolution investigations of short-term changes in ciliate abundance and community composition are highly desired to gain insights into the intricate temporal dynamics of ciliates and their driving factors.

Recent years have seen an increase in the use of molecular approaches, e.g., sequencing of marker genes, such as the SSU rRNA gene, to explore the diversity and distribution of ciliates from a variety of marine environments (Grattepanche et al., 2014; Santoferrara et al., 2016; Sun et al., 2017, 2019, 2020, 2021; Zhao et al., 2017). However, sequencing-based methods cannot offer direct data on the abundance and biomass of ciliates, which is necessary for inferring their potential ecological functions (Santoferrara et al., 2016). Furthermore, due to the limitations of the reference database used to assign taxonomic identities to the retrieved sequences, many sequences cannot be confidently classified at the species level. Ciliates have been reported to feed on prey ca. 5–30% of their length and most efficiently on prey ca. 25% of their oral diameter (Dolan et al., 2013). Thus, ciliate size and oral diameter were considered to represent conservative taxonomic/functional traits (Dolan et al., 2013). Again, such information is accessible exclusively through morphology-based techniques, such as the quantitative protargol staining method (Meng et al., 2018; Sun et al., 2019; Yang et al., 2020; Gu et al., 2021; Huang et al., 2021). Details on the variations of ciliate size and oral diameter, abundance, biomass, and community composition during short-term time-series sampling may provide insights into their ecological niche and roles.

Thus, to explore the dynamic patterns of ciliates and to elucidate the environmental driving factors on a short time scale, a high frequency sampling strategy was conducted hourly for six non-continuous days in the high salinity waters (>26) of the Jiulong River estuary in southeast China. The Jiulong River estuary is a typically subtropical estuary (Supplementary Figure S1) that receives substantial quantities of nutrient-rich freshwater discharge (1.17 × 10^10^ m^3^ per year) from the Jiulong River (Huang and Hong, 1999). The river is mostly fed by the summer monsoon, which occurs between April and July, and there is no dam upstream to regulate the river’s flow (Huang and Hong, 1999). The suspended particles and nutrients, particularly NO_3_-N and soluble reactive phosphorus, in the Jiulong River estuary originated primarily from the Jiulong River (Yan et al., 2012). Previous studies have reported the distribution of polycyclic aromatic hydrocarbon (PAH) degrading bacteria, the seasonal dynamics of bacterial communities, the biogeographical patterns of archaea, the spatial/temporal distribution of microbial eukaryotes, and the seasonal changes of ciliates along salinity gradients in the Jiulong River estuary (Tian et al., 2002; Hu et al., 2015; Sun et al., 2017; Kong et al., 2019; Wang et al., 2019). However, no investigations of the short-term variations in ciliate assemblages have been conducted in this area. Our work aims to (i) explore changes in the abundance, biomass, size, oral diameter, and community composition of ciliates at a high resolution and (ii) gain insight into the coupling of ciliates and environmental parameters.

## Materials and Methods

### Sample Collection

The sampling site (S03, 118.031°E, 24.429°N) is located near the mouth of the Jiulong River and is significantly influenced by river discharge and seawater intrusion (Wang et al., 2019). Hourly samples were collected from 8:30 a.m. to 4:30 p.m. on April 3 and from 8:30 a.m. to 5:30 p.m. on April 5, 7, 9, 11, and 16, 2016. A 5 L polycarbonate bottle was used to collect seawater samples at a depth of ca. 0.5 m. Samples for phytoplankton pigment analysis were collected by filtering 500 ml of seawater through a 0.7 μm pore size, 47 mm GF/F (Gleman) glass fiber filter and immediately frozen in liquid nitrogen. Nutrient samples were filtered using the same filter and stored at –20°C until analysis. Two ml of seawaters were pre-filtered through a 20-μm mesh and fixed with ice-cold glutaraldehyde (0.5% final concentration) at room temperature for 15 min in the dark, then flash-frozen in liquid nitrogen to determine the abundance of picoplankton, including pigmented picoeukaryotes (PPEs), heterotrophic prokaryotes (HPs), *Synechococcus*, and viral-like particles (VLPs). For ciliate identification and enumeration, 500 ml of seawaters were fixed with Bouin’s solution (10% final concentration) with the addition of ice-cold acetic acid (1% final concentration) before fixation and stored in the dark at 20°C until further analysis.

### Determination of Environmental Parameters

Temperature, salinity, and dissolved oxygen (DO) were determined *in situ* using a YSI Pro2030 (YSI Life Sciences, Yellow Springs, OH, United States). The inorganic nutrients, including phosphate, silicate, nitrite, and nitrate, were measured using a Seal AA3 auto-analyzer (Bran-Luebbe, GmbH, Delavan, WI, United States).

Phytoplankton pigments were determined following Huang et al. (2010). To summarize, phytoplankton pigments were extracted using *N,N*-dimethylformamide (Furuya and Marumo, 1983) and analyzed with a high performance liquid chromatography (HPLC) system (Agilent Series 1100, Agilent Technologies, Santa Clara, CA, United States) equipped with a 3.5-mm Eclipse XDB C8 column (100 × 4.6 mm; Agilent Technologies, Santa Clara, CA, United States) following Mantoura and Llewellyn (1983) and Van Heukelem and Thomas (2001).

The abundance of picoplankton and VLPs was determined using flow cytometers (Epics Altra II, Beckman Coulter, Brea, CA, United States, and BD Accuri C6, Franklin Lake, NJ, United States). Subsamples were stained with SYBR Green I (Molecular Probe, Eugene, OR, United States) according to the published protocol before assessing viral and HPs abundance (Marie et al., 1999). Fluorescent beads (Molecular Probes) with a diameter of 1 μm were added as an internal standard. *Synechococcus* and PPEs were identified without staining by their pigment fluorescence (Marie et al., 1999). The FlowJo vX.0.7 software (Tree Star, Ashland, OR, United States) was used to process all the data generated by flow cytometers.

### Ciliate Identification and Enumeration

To determine the abundance, size, oral diameter, and species identity of ciliates, the quantitative protargol stain method was employed (Montagnes and Lynn, 1987). Briefly, the fixed samples (500 ml each) were filtered through one or two 0.8 μm pore size, cellulose nitrate filters (Whatman, Florham Park, NJ, United States), embedded in a thin layer of agar, stained with protargol, dehydrated with isopropanol and xylene solutions, and mounted using neutral balsam on microscopic slides. Each slide was thoroughly scanned at ×400 magnification, and all ciliates were identified and counted using a compound microscope (Olympus BX50). Ciliates were identified according to Montagnes and Lynn (1991), Strüder-Kypke and Montagnes (2002), and Xu et al. (2009). The carbon biomass of ciliates was calculated using simple geometric volume (Hillebrand et al., 1999) multiplied by a conversion factor of 0.19 pg C μm^–3^ (Putt and Stoecker, 1989).

### Data Analysis

All statistical analyses were conducted using R 4.0.3. The “*GGally*” package in R based on Spearman correlations was used to analyze and visualize the association between ciliate abundance/biomass and environmental parameters [water temperature, salinity, DO, phosphate, silicate, nitrite, nitrate, *Synechococcus*, HPs, PPEs, high fluorescence content virus (HFV), and low fluorescence content virus (LFV)]. Additionally, a generalized linear model (GLM) based on Gaussian distributions was used to identify highly correlated variables and determine the best fit. The variance inflation factor (VIF) was used to test multicollinearity, and variables with a VIF > 10 were eliminated from the model (Graham, 2003). The GLM results aided in testing a set of hypothesized pathways that served as a framework for developing a multivariate model in which all parameters might function as endogenous (dependent) or exogenous (predictor) variables. We used confirmatory path analysis, a form of structural equation modeling (SEM), to assess possible relationships between environmental parameters and ciliate abundance (Shipley, 2000). SEM was calculated using the “*lavaan*” package in R (Rosseel, 2012), and model fit was tested by the metrics of Chi-square value (χ^2^), comparative fit index (CFI), and root square mean error of approximation (RMSEA) (Schermelleh-Engel and Moosbrugger, 2003). The total number of samples for the model was 59.

A principal coordinate analysis (PCoA) plot based on Bray Curtis dissimilarities was used to depict the ordination of the ciliate community with the “*vegan*” package in R (Oksanen et al., 2010). Additionally, the Mantel test was performed with the same R package to explore the correlations between ciliate communities and environmental parameters.

## Results

### Environmental Parameters

**FIGURE 1:**
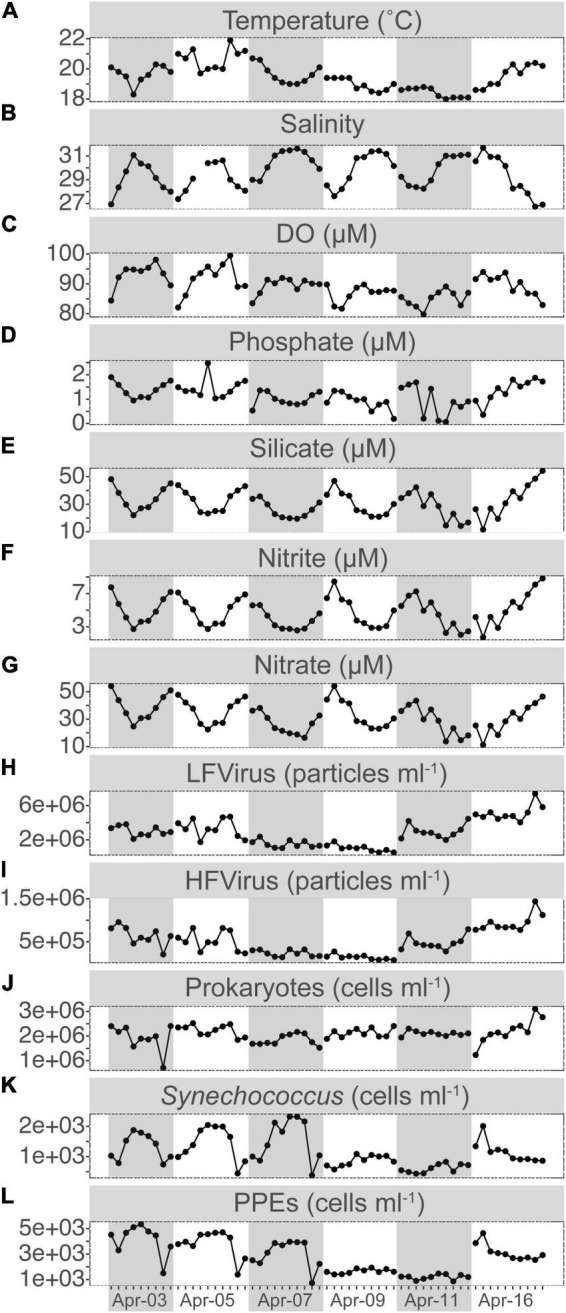
Short-term time series of the dynamics for temperature **(A)**, salinity **(B)**, dissolved oxygen (DO, **C**), phosphate **(D)**, silicate **(E)**, nitrite **(F)**, and nitrate **(G)** concentrations, LFvirus (low-fluorescence content virus, **H**), HFvirus (high-fluorescence content virus, **I**), heterotrophic prokaryotes **(J)**, *Synechococcus* **(K)**, and PPEs (pigmented picoeukaryotes, **L**) abundance. Gray or non-gray shadow represented different sampling days.

The water temperature at S03 ranged between 18.0 and 21.9°C, with a daily variation of ca. 2°C. Relatively modest salinity changes between 26.74 and 31.68 were recorded, which exhibited a significant negative correlation with temperature (*r* = –0.59, *p* < 0.001) (Figure 1 and Supplementary Figure S2). Salinity was typically highest in the morning and evening and lowest at noon, except on April 16. The average concentrations of phosphate, silicate, nitrite, and nitrate were 1.17 ± 0.48, 30.89 ± 9.63, 4.74 ± 1.78, and 32.34 ± 10.51 μM, respectively, and all of them had a significantly inverse relationship with salinity (*r* = –0.60, *p* < 0.01; *r* = –0.95, *p* < 0.001; –0.95, *p* < 0.001; *r* = –0.91, *p* < 0.001) and covaried with temperature (*r* = 0.56, *p* < 0.001; *r* = 0.60, *p* < 0.001; *r* = 0.52, *p* < 0.001; *r* = 0.59, *p* < 0.001) (Figure 1 and Supplementary Figure S2).

PPEs were the dominant picophytoplankton throughout our study period, with a mean abundance of 2.80 ± 1.34 × 10^3^ cells ml^–1^, about twice that of *Synechococcus*. During the first 3 days, PPEs and *Synechococcus* both showed relatively comparable patterns of variation in abundance, with a peak at noon and a relative decrease in the morning and evening (Figure 1). Compared to PPEs and *Synechococcus*, the patterns of HPs, HFVs, and LFVs abundance did not exhibit apparent daily regularity (Figure 1). The abundances of HFVs and LFVs increased from 7.34 × 10^4^ and 5.27 × 10^5^ cells ml^–1^ (17:30 on Apr 9) to 1.44 × 10^6^ and 7.38 × 10^6^ cells ml^–1^ (16:30 on Apr 16) (ca. a 20-fold increase for HFVs and a 14-fold increase for LFVs, respectively), whereas the HPs abundance increased from 7.18 × 10^5^ cells ml^–1^ (15:30 on Apr 3) to 3.10 × 10^6^ cells ml^–1^ (16:30 on Apr 16, ca. 30-fold increase).

### Ciliate Abundance, Biomass, Cell Size, Oral Diameter, and Correlations With Environmental Parameters

**FIGURE 2:**
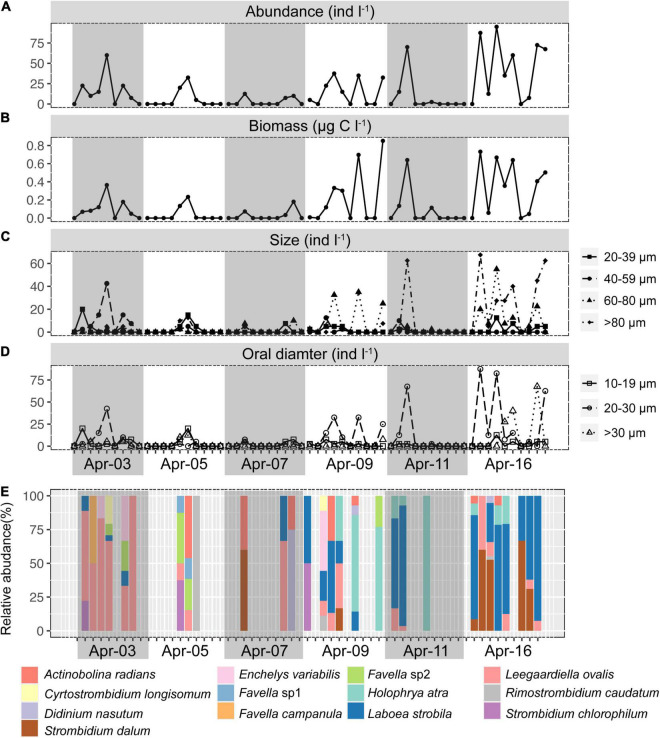
Short-term time series of the abundance **(A)**, biomass **(B)**, size structure **(C)**, oral diameter structure **(D)**, and community composition of ciliates **(E)**. Gray or non-gray shadow represented different sampling days.

Ciliates were a modest component of the counted microbial plankton, with an abundance of 4–7 orders of magnitude lower than picoplankton (Figures 1, 2A). Ciliates were not found in about half (31/59) of the samples. Ciliate abundance averaged 15.21 ± 24.30 cells l^–1^ and varied significantly (Figures 2A,B) throughout the day and sampling period, peaking at 95 cells l^–1^ at 11:30 on Apr 16. When all time-series data were integrated, ciliate abundance showed only significant correlations with biotic factors, including HPs (*r* = 0.26, *p* < 0.05), HFVs (*r* = 0.41, *p* < 0.01), and LFVs abundance (*r* = 0.38, *p* < 0.01). By contrast, no significant correlations (*p* > 0.05) between ciliate abundance and abiotic factors were found. Higher biomass of ciliates was observed in the final 3 days (0.22 ± 0.29 μg C l^–1^) than in the first 3 days (0.05 ± 0.09 μg C l^–1^), and the maximum biomass (0.85 μg C l^–1^) was found at 17:30 on Apr 9. To assess changes in the cell size and oral diameter structure, ciliates were divided into four size fractions (20–39, 40–59, 60–80, and >80 μm) and four oral diameter categories (10–19, 20–30, and >30 μm). The ciliate community was dominated by the 40–59 (29.81%) and 60–80 μm (43.45%) cell size fractions, as well as the 20–30 μm (63.32%) oral diameter fractions (Figures 2C,D).

**FIGURE 3:**
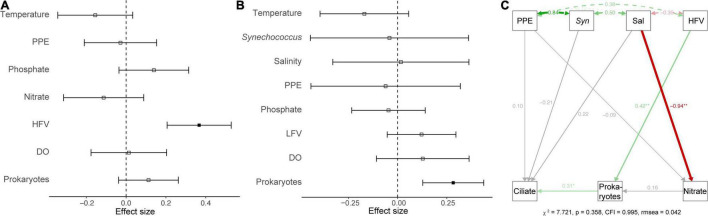
A generalized linear model showed the effect of environmental variables on the abundance **(A)** and biomass **(B)** of ciliates. Significant (*p* < 0.05) and non-significant (*p* > 0.05) effects were marked by the solid or non-solid square dots, respectively. **(C)** Structural equation model that describes potential direct impacts of environmental variables (PPEs, *Syn*, salinity, HFV, heterotrophic prokaryotes, and nitrite) on ciliate biomass. The solid green and red arrows indicated significant (*p* < 0.05) positive and negative associations, respectively. The gray arrows indicated that the correlations were non-significant (*p* > 0.05). Double-headed arrows represented covariances. Path coefficients are displayed next to arrows and represent the expected change in the response given a one-unit change in the predictor given the other variables.

Generalized linear models (GLM) were used to determine the main and interactive effects and effect sizes of environmental parameters on ciliate abundance and biomass. The results indicated that environmental factors (e.g., temperature and salinity) had an insignificant effect on the ciliate abundance and biomass (*p* > 0.05). However, biotic variables (e.g., HFVs and HPs) were significant covariates (*p* < 0.05) of ciliate abundance and biomass (Figures 3A,B). A path analysis was also used to build a multivariate model that could be used as a framework for all parameters to function as dependent or predictor variables (Figure 3C). The final model confirmed that salinity had a significant direct effect on nitrate concentration (*r* = –0.94, *p* < 0.01) but not on ciliate abundance (*r* = 0.22, *p* > 0.05). Significant pathways (*r* = 0.31, *p* < 0.05) were discovered between ciliates from HPs that are significantly influenced by HFVs (*r* = 0.42, *p* < 0.01) (Figure 3C). Overall, the resulting SEM fit our data well (χ2 = 7.721, *p* = 0.358, CFI = 0.995, rmsea = 0.042), indicating the potential control mechanism of ciliate biomass.

### The Composition of Ciliate Communities and Their Relationships With Environmental Variables

**FIGURE 4:**
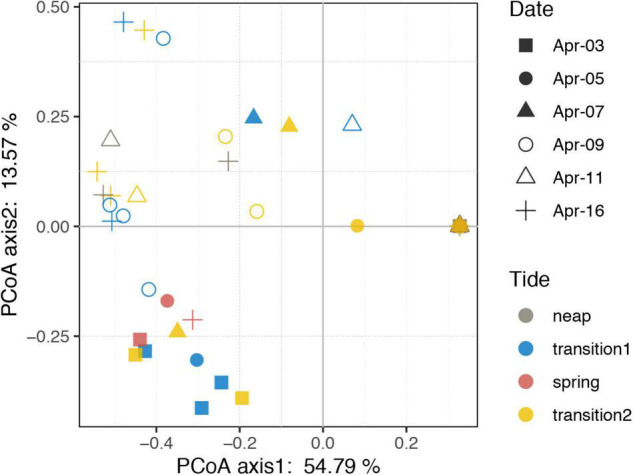
Principal coordinate analysis plot based on Bray–Curtis dissimilarity of the ciliate community. Neap, transition, and spring tide were distinguished based on the distinct variations of tidal range. Transition 1 and 2 represents the transition from neap to spring and from spring to neap tides, respectively.

Thirteen species were retrieved during the study period (Figure 2E and Supplementary Figure S3). *Leegaardiella ovalis* (ca. 22.41% of overall abundance), *Laboea strobila* (ca. 27.98%), *Strombidium dalum* (ca. 10.19%), and *Holophrya atra* (ca. 12.04%) were the most abundant species. Generally, the ciliate community exhibited no clear pattern of occurrence throughout each day. There were differences between different days. For example, the first day was dominated by *L. ovalis* (ca. 66.67%), whereas the last 3 days were dominated by *L. strobila* (ca. 44.22%) (Figure 3C). In the PCoA based on Bray Curtis distance, ciliate communities were fragmented and were not grouped according to the sampling time or tides (i.e., spring, neap, and transitional periods), indicating that the community composition fluctuated significantly within and between days (Figure 4). The Mantel test revealed that the ciliate community was significantly correlated with Lutein (*r* = 0.438, *p* < 0.001) and Neoxanthin (*r* = 0.312, *p* = 0.008). Phosphate and PPEs were also found to be significantly correlated with the ciliate community, but with rather low *r* values (*r* = 0.210, *p* = 0.028 for phosphate and *r* = 0.163, *p* = 0.037 for PPEs, respectively). Temperature (*r* = –0.01, *p* = 0.507) and salinity (*r* = –0.05, *p* = 0.767) had no significant effects on the ciliate community (Table 1).

**TABLE 1:** Mantel test comparison between ciliate community variability (measured as Bray–Curtis dissimilarity) and environmental parameters.

Environmental parameters		*r*	*p*
Temperature		0.016	0.395
Salinity		–0.083	0.826
DO		0.098	0.135
DIP		**0.210**	**0**.**028**
SiO_4_^–^		–0.089	0.808
NO_2_^–^ + NO_3_^–^		–0.064	0.764
NO_2_^–^		–0.058	0.741
NO_3_^–^		–0.066	0.769
Microbial abundance	*Synechococcus*	0.085	0.184
	HPs	–0.002	0.471
	PPEs	**0.163**	**0**.**037**
	Viruses	0.005	0.449
	LNVs	0.007	0.440
	HNVs	0.004	0.458
Phytoplankton pigments	Chlorophyll c3	–0.032	0.586
	Chlorophyllide *a*	0.036	0.366
	Chlorophyll c2 + c1	0.130	0.129
	Peridinin	0.039	0.351
	Fucoxanthin	0.013	0.427
	Neoxanthin	**0.312**	**0**.**008**
	Prasinoxanthin	0.075	0.199
	19′-hexanoyloxyfucoxanthin	–0.014	0.512
	Violaxanthin	0.121	0.104
	Diadinoxanthin	0.095	0.170
	Alloxanthin	0.113	0.157
	Zeaxanthin	–0.051	0.662
	Lutein	**0.438**	**0**.**000**
	Chlorophyll *b*	0.122	0.123
	Chlorophyll *a*	0.132	0.139
	α-carotene	0.150	0.112
	β-carotene	0.090	0.211

*Numbers in bold indicate statistically significant results. PPEs, pigmented picoeukaryotes; LFV, low-fluorescence virus; HFV, high-fluorescence virus; DO, dissolved oxygen; HPs, heterotrophic prokaryotes.*

## Discussion

To our best knowledge, this is the first study to explore the dynamics of ciliates using the quantitative protargol stain approach over a short-term and high-frequency time series (i.e., hourly sampling). At S03, the coupling of temperature, salinity, and nutrients was observed (Figure 1), which closely mirrored the dilution effect pattern observed in estuarine environments where nutrients from eutrophic freshwater were diluted by oligotrophic offshore seawater. Our data showed unequivocally that a high-frequency sampling strategy could be used to detect a wide range of temporal changes in ciliate assemblages in the Jiulong River estuary. We emphasized that in subtropical estuarine environments, ciliates (abundance, biomass, size and oral diameter structure, and community composition) exhibited dramatic variations within a few days, even a few hours. Using Spearman correlation, GLM, path analysis, and mantel test, we found that biological factors (PPEs, HPs, viruses, and phytoplankton pigments) were the driving factors of ciliate variations in the waters with high nutrient input and salinity (26.7–31.7) of the Jiulong River estuary.

### High Dynamics of Ciliate Assemblages

In general, the abundance of ciliates varied substantially across the study period at the Jiulong River estuary sampling site, with a narrow gradient of high estuarine salinities. In comparison to other coastal estuaries and bays worldwide (Chiang et al., 2003; Strom et al., 2007; Tsai et al., 2011; Haraguchi et al., 2018; Loìpez-Abbate et al., 2019; Yang et al., 2020; Gu et al., 2021), the abundance of ciliates was comparatively lower, ranging between 0 and 95 cells l^–1^, although this was within the range reported in some literature (Santoferrara and Alder, 2009; Santoferrara et al., 2010). Additionally, due to the small number of sampling stations (1 station) and the short sampling period (6 days), only 13 ciliate species were recovered, far fewer than in previous large-area sampling studies in the estuary (Tsai et al., 2011; Yang et al., 2020; Gu et al., 2021). Long-term time series investigations in estuarine ecosystems have revealed seasonal changes in ciliate abundance, biomass, size, and community composition (Tsai et al., 2011; Haraguchi et al., 2018). Our study further revealed that the abundance, biomass, size, oral diameter, and community composition of ciliates changed rapidly and irregularly within a few days or even a few hours in the estuary (Figure 2), and these dynamics influenced prey populations (e.g., HPs, PPEs, and *Synechococcus*) via top–down control. For instance, changes in the oral diameter and community structure of ciliate assemblages may alter prey composition, as various species have different grazing preferences (Gong et al., 2016), and ciliates feed most efficiently on prey ca. 25% of their oral diameter (Dolan et al., 2013). Additionally, the dominance of the ciliate community shifted from *Leegaardiella ovalis* on Apr 3 to *Laboea strobila* on Apr 16, indicating that the function of the ciliate assemblages may transition from heterotrophic to phototrophic/mixotrophic, thus potentially affecting the primary productivity of the system.

### Environmental Factors Shaping the Abundance, Biomass, and Community Composition of Ciliates

In estuarine ecosystems, salinity is commonly considered a crucial factor in shaping microbial populations (Campbell and Kirchman, 2013; Xia et al., 2017), including ciliates (Elloumi et al., 2006; Yang et al., 2020; Gu et al., 2021). In this study, *Synechococcus* and HPs abundances increased with salinity (Supplementary Figure S2, *r* = 0.50, *p* < 0.001; *r* = 0.34, *p* < 0.05, respectively), confirming earlier findings (Jochem, 2003; Zhang et al., 2013; Chen et al., 2019). However, the observed ciliate abundance and biomass did not follow this trend (Supplementary Figure S2), exhibiting substantial changes within hours or days (Figure 2). Our results showed that salinity had only a minor effect on ciliate abundance and biomass (Figures 3A,B). During the study period, salinity fluctuated slightly (26.7–31.0). Because sampling a high salinity range was not the focus of this work, the “real” effect of salinity on estuarine ciliate communities cannot be detected by the present dataset.

Ciliates have been identified as the primary bacterial grazers and are expected to consume up to 100% of the estimated protozoan bacterivory in coastal waters (Sherr and Sherr, 1987; Zhao et al., 2020). In the present study, SEM analysis revealed a significant path from HFVs to HPs and then to ciliates, implying changes in ciliate biomass over a short time in the Jiulong River estuary may be attributed to changes in food supply (HPs). Chen et al. (2019) highlighted the importance of virus-HPs interactions in microbial dynamics (i.e., bacterial production and community composition) in the Jiulong River estuary. We further discovered that virus-HPs interactions might play a role in regulating ciliate biomass via bottom-up control in the Jiulong River estuary. HPs are highly sensitive to changes in ionic strength, and variation in salinity (26.7–31.7) during the tidal cycle may result in a shift in the physiological stress on metabolic processes of HPs (Kukkaro and Bamford, 2009; Wei et al., 2019). As with HPs, the realized niche of viruses would be confined by salinity, as salt stress has been shown to directly affect their survival and ability to infect (Wei et al., 2019). Ciliates appeared to be less salinity-sensitive than HPs and viruses because they expanded across a wide range of salinities (Li et al., 2018, 2019). However, due to a lack of data on nanoflagellates and copepods, which are commonly acknowledged as the food and grazers of ciliates, it was unknown whether nanoflagellates and copepods affected ciliate abundance, biomass, and community composition in the Jiulong River estuary during a short period. Overall, our study found that in the high salinity waters (26.7–31.7) of the Jiulong River estuary, salinity has a minor direct regulatory effect on ciliate abundance and biomass. In contrast, virus-HPs interactions drove the changes in ciliate biomass (Figure 3).

Additionally, phytoplankton pigments including Lutein (*r* = 0.438, *p* < 0.001) and Neoxanthin (*r* = 0.312, *p* = 0.008) were found to be significantly correlated with ciliate community composition (Table 1). In the estuary, Lutein and Neoxanthin were mostly found in chlorophytes, serving as food sources for ciliates (Ansotegui et al., 2003). Lutein and Neoxanthin accounted for ca. 0–58.8% of total phytoplankton pigments, with an average of ca. 20% (Supplementary Figure S4). It has been reported that protistan grazers can preferentially hunt certain groups of phytoplankton (Li et al., 2021). Thus, the composition of ciliate communities may be directly altered by the phytoplankton communities through grazing. Meanwhile, ciliate communities may be altered due to predation on nanoflagellate assemblages, which typically feed on phytoplankton. Additionally, it has been proposed that phytoplankton can mobilize organic nutrients, affecting the organic nutrients available to heterotrophic prokaryotes and thereby affecting the grazers, i.e., nanoflagellates and ciliates. This “indirect effect” of phytoplankton-organic nutrients-heterotrophic prokaryotes-grazers may also account for the correlations found between phytoplankton pigments and ciliate communities in this study (Fenchel, 2008). Phosphate was the third factor that significantly correlated with ciliate community composition (Table 1, *r* = 0.17, *p* = 0.045, Mantel test), consistent with a recent report conducted in the Pearl River estuary (Gu et al., 2021). We surmised that it was owing to the dominance of *L. strobila*, an autotrophic/mixotrophic ciliate species, that might require dissolved inorganic phosphate (DIP) for growth (McManus and Fuhrman, 1986).

## Conclusion

In summary, our work established that the abundance, biomass, cell size and oral diameter structure, and community composition of ciliates in high salinity waters (>26) of the Jiulong River estuary altered significantly and irregularly during a short period (i.e., both hourly and daily). Biotic factors (HFVs and HPs) were strongly associated with ciliate abundance and biomass rather than abiotic factors. Further analysis showed that the path from salinity to HPs and viruses and finally to ciliates might account for the dynamics of ciliate biomass. In contrast, the ciliate community was primarily shaped by phytoplankton pigments, including Neoxanthin and Lutein, followed by phosphate and PPEs. Our results bridged the gap in our understanding of ciliate dynamic patterns on a short time scale and identified the potential forcing environmental factors in the high salinity estuarine waters of a subtropical estuary.

## Data Availability Statement

The raw data supporting the conclusions of this article will be made available by the authors, without undue reservation.

## Author Contributions

BG: formal analysis and writing – original draft. HH and YW: formal analysis. YZ: investigation and formal analysis. RL, LW, JS, and JW: investigation. RZ: writing – review and editing. NJ: writing – review and editing, funding acquisition. DX: conceptualization, formal analysis, writing – review and editing, and supervision. All authors: contributed to the article and approved the submitted version.

## Conflict of Interest

The authors declare that the research was conducted in the absence of any commercial or financial relationships that could be construed as a potential conflict of interest.

## Publisher’s Note

All claims expressed in this article are solely those of the authors and do not necessarily represent those of their affiliated organizations, or those of the publisher, the editors and the reviewers. Any product that may be evaluated in this article, or claim that may be made by its manufacturer, is not guaranteed or endorsed by the publisher.
