# Supplementary material for: High Dynamics of Ciliate Community Revealed via Short-Term, High-Frequency Sampling in a Subtropical Estuarine Ecosystem
Source: Front Microbiol. 2022 Feb 7;13:797638. doi: 10.3389/fmicb.2022.797638 (PMC8858835; doi:10.3389/fmicb.2022.797638)
Supplement: Supplementary file 1 [file Data_Sheet_1.DOCX]

***Supplementary Material***

**Supplementary Table**

**Supplementary Table 1.** All samples were divided into four groups (Spring, neap, transition1 and transition2) according to the tidal height when sampling. Transition 1 and 2 represents the transition from neap to spring and from spring to neap tides, respectively.

| Spring tide | Neap tide | Transition1 | Transition2 |
| --- | --- | --- | --- |
| 4.3-12 | 4.3-16 | 4.3-8 | 4.3-13 |
| 4.5-13 | 4.7-8 | 4.3-9 | 4.3-14 |
| 4.7-13 | 4.7-9 | 4.3-10 | 4.3-15 |
| 4.9-15 | 4.9-9 | 4.3-11 | 4.5-14 |
| 4.11-16 | 4.11-10 | 4.5-8 | 4.5-15 |
| 4.16-10 | 4.16-15 | 4.5-9 | 4.5-16 |
|  | 4.16-16 | 4.5-10 | 4.5-17 |
|  |  | 4.5-11 | 4.7-15 |
|  |  | 4.5-12 | 4.7-16 |
|  |  | 4.7-10 | 4.7-17 |

**Supplementary Figures**


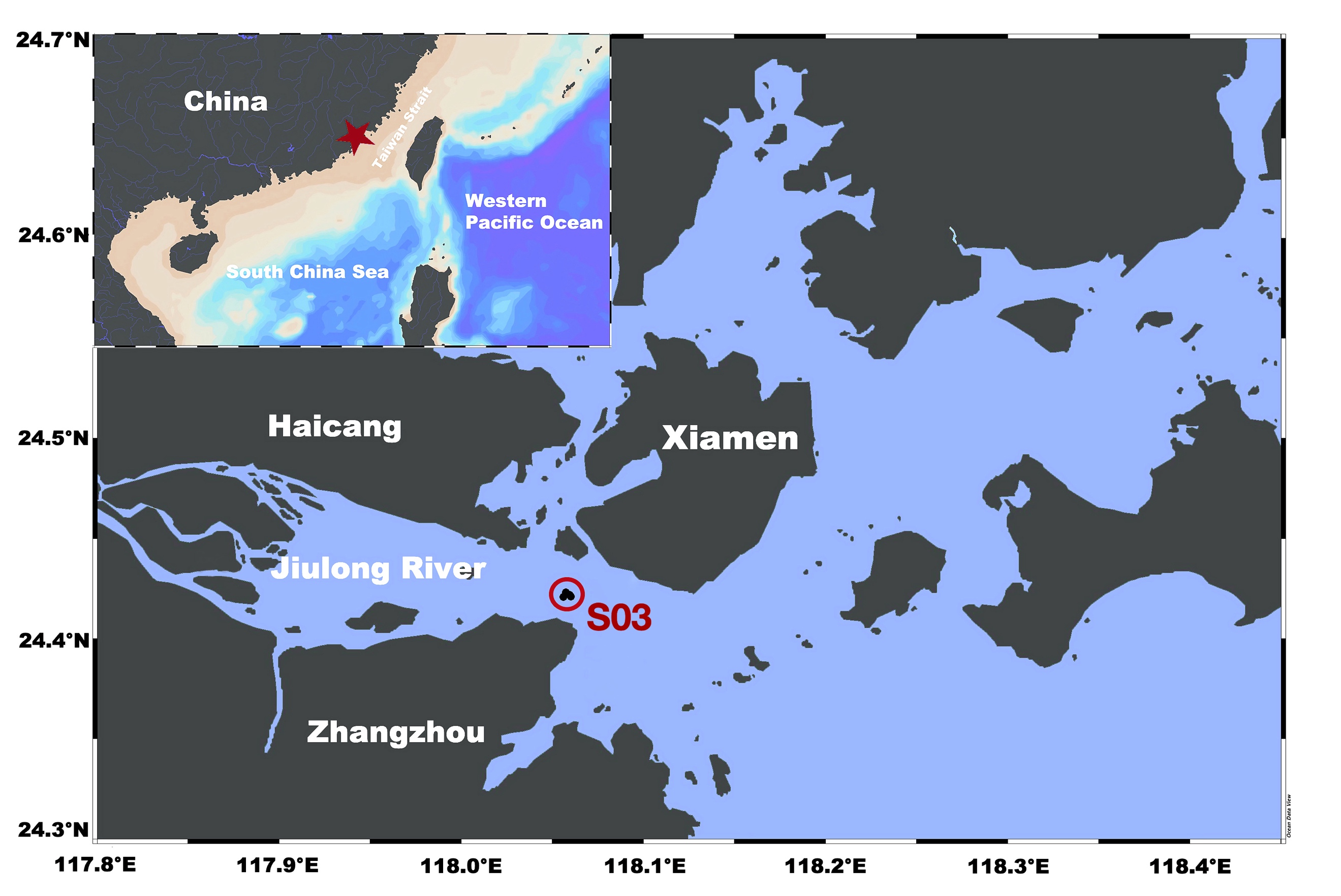


**Supplementary Figure 1.** The sampling station (S03) in the JRE.


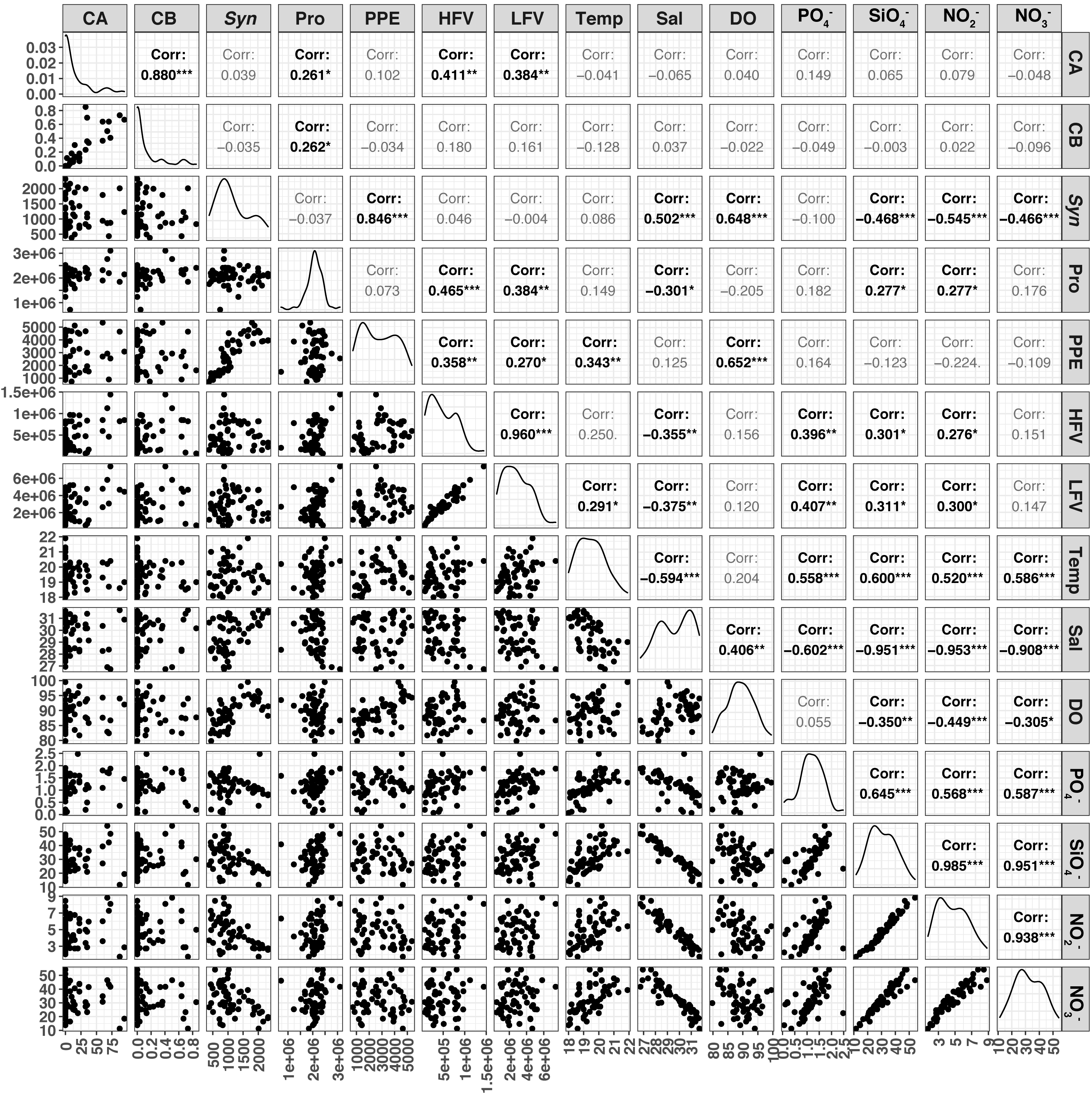


**Supplementary Figure 2.** Scatter plot matrix (below the diagonal), histograms (diagonal) and Spearman correlation coefficient (above the diagonal) among ciliate abundance (CA), biomass (CB), *Synechococcus* (*Syn*), prokaryotic (Pro), pigmented picoeukaryote (PPE), high-fluorescence viral (HFV), low-fluorescence viral (LFV) abundance with temperature (Temp), salinity (Sal), dissolved oxygen (DO), phosphate (PO_4_^-^), silicate (SiO_4_^-^), nitrite (NO_2_^-^) and nitrate (NO_3_^-^) concentration. Significant correlations are bold and indicated with (*). *p < 0.05; **p < 0.01; ***p < 0.001.


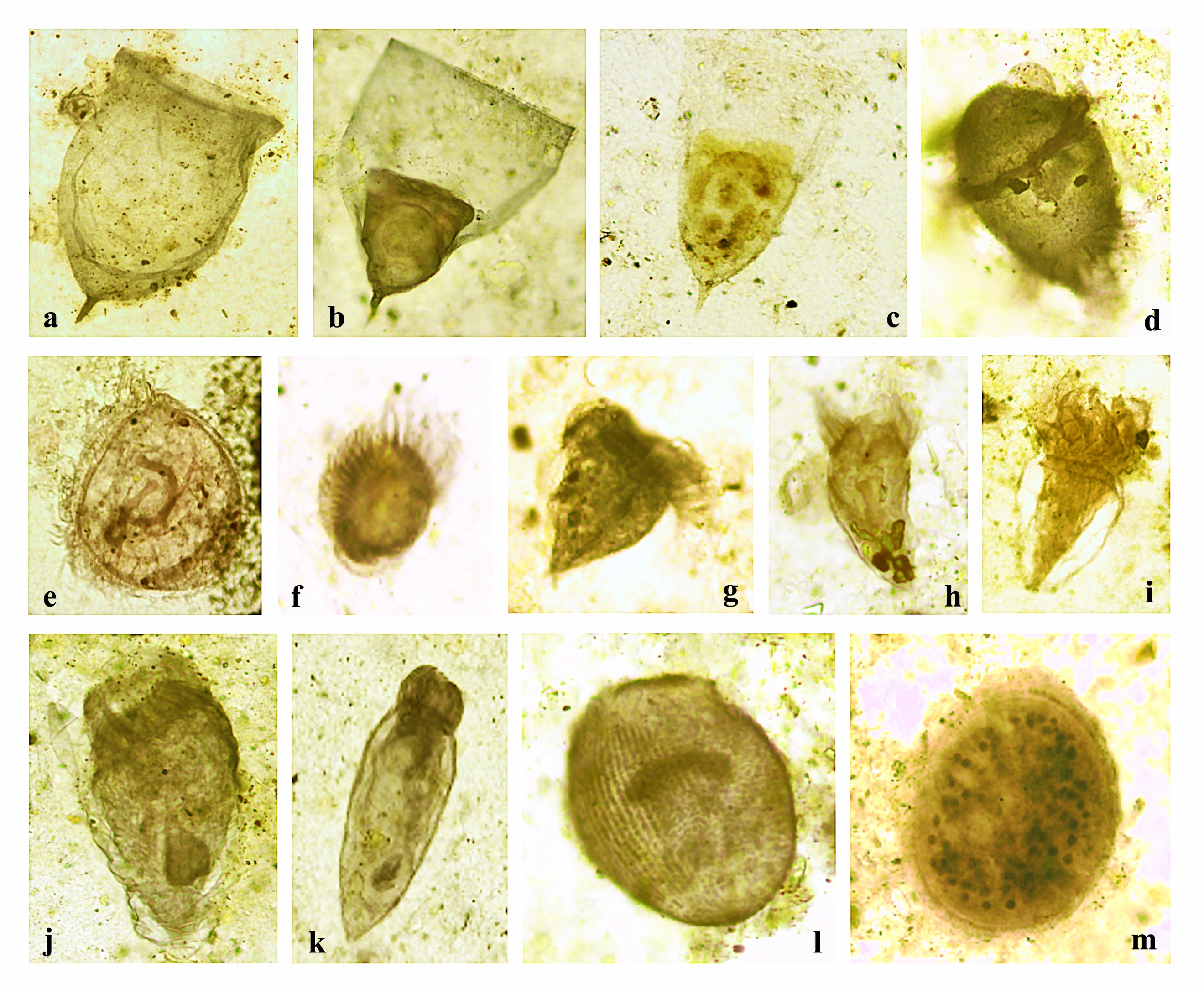


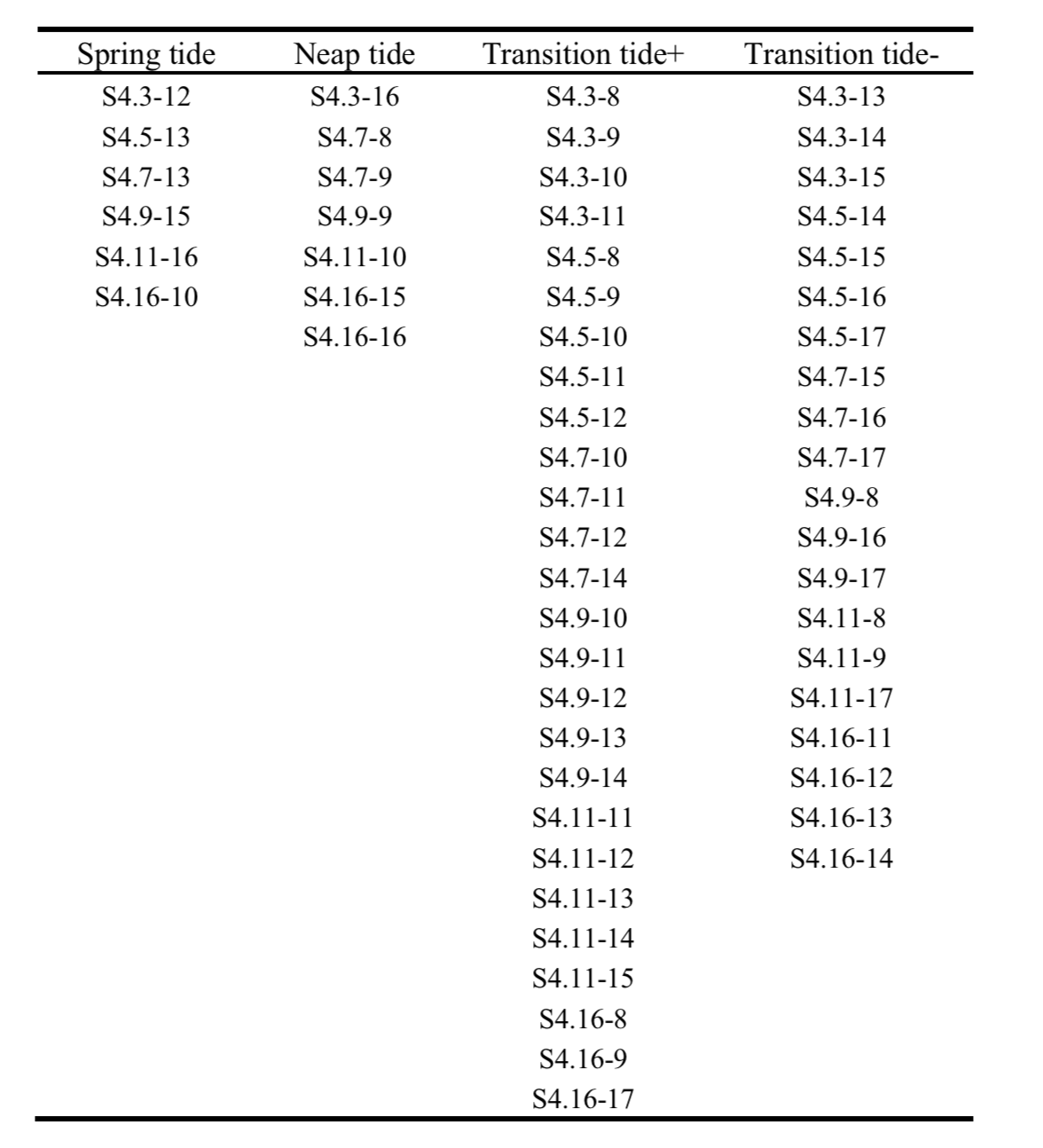
**Supplementary Figure 3.** Photomicrographs of planktonic ciliates in the S03 over short-term time series revealed by quantitative protargol staining method. (a) *Favella sp1*, (b) *Favella sp2*, (c) *Favella campanula*, (d) *Didinium nasutum*, (e) *Actinobolina radians*, (f) *Leegaardiella ovalis*, (g) *Rimostrombidium caudatum*, (h) *Strombidium chlorophilum*, (i) *Strombidium dalum*, (j) *Laboea strobila*, (k) *Cyrtostrombidium longisomum*, (l) *Enchelys variabilis*, (m) *Holophrya atra*.


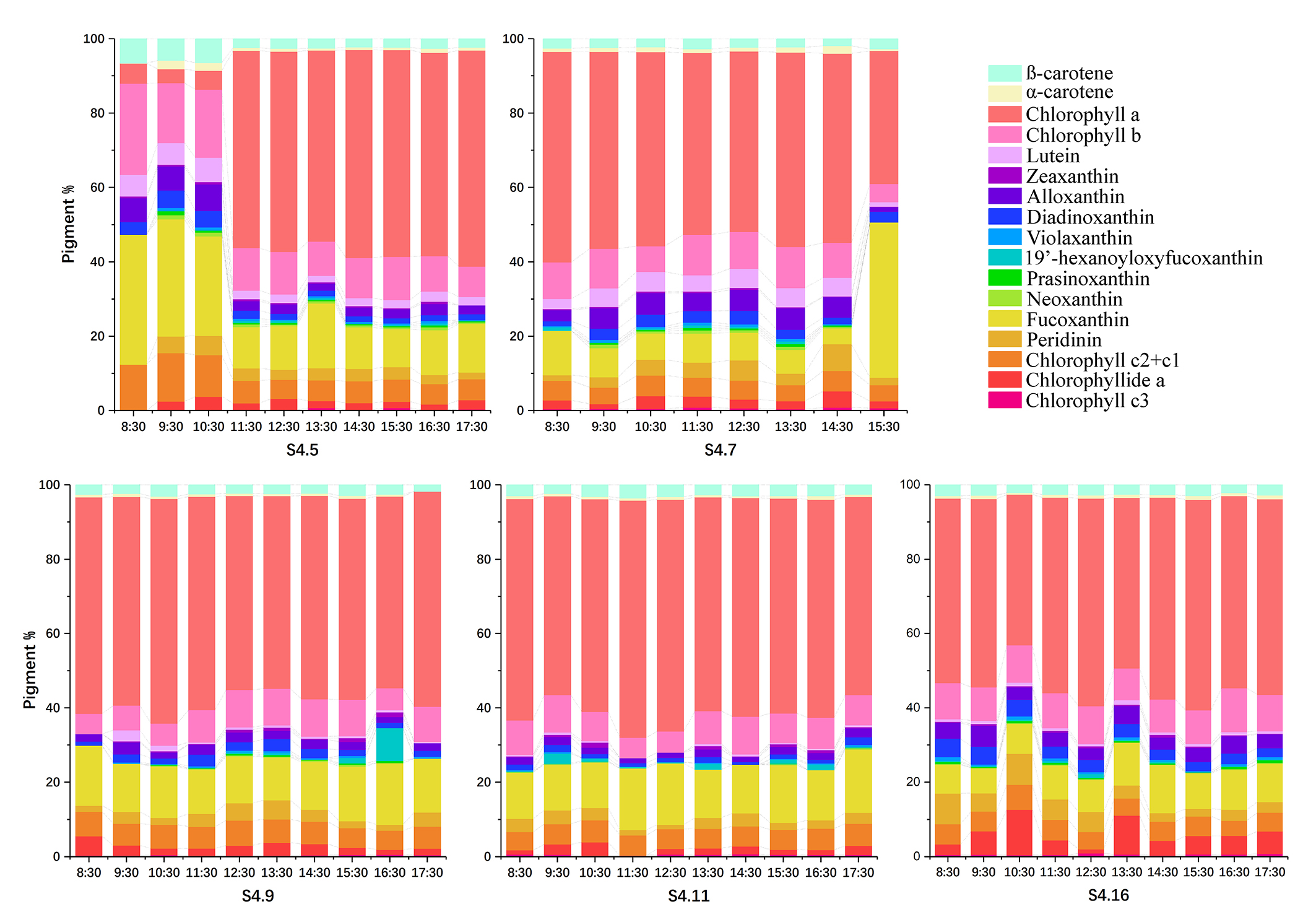


**Supplementary Figure 4.** Proportions of different types of phytoplankton pigments in each sampling day.


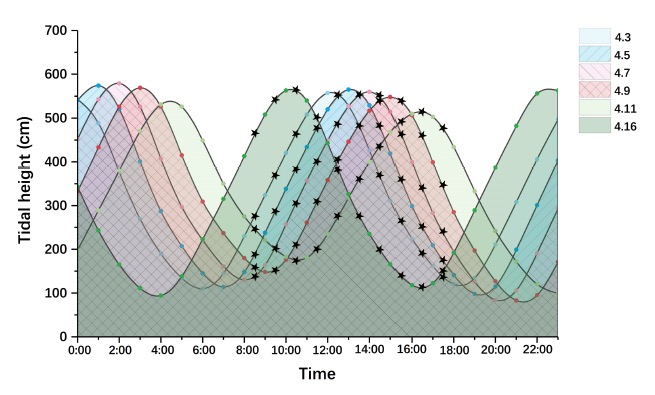


**Supplementary Figure 5.** The dynamics of tidal height over spring-neap tidal cycle in the JRE in April, 2016. The sapling time were labeled with stars.
